# Brain Transcriptome Profiling Analysis of Nile Tilapia (*Oreochromis niloticus*) Under Long-Term Hypersaline Stress

**DOI:** 10.3389/fphys.2018.00219

**Published:** 2018-03-15

**Authors:** Yan Liu, Erchao Li, Chang Xu, Yujie Su, Jian G. Qin, Liqiao Chen, Xiaodan Wang

**Affiliations:** ^1^Department of Aquaculture, College of Marine Sciences, Hainan University, Haikou, China; ^2^Laboratory of Aquaculture Nutrition and Environmental Health, East China Normal University, Shanghai, China; ^3^School of Biological Sciences, Flinders University, Adelaide, SA, Australia

**Keywords:** salinity, immunity, osmoregulation, transcriptome, gene, pathway

## Abstract

The fish brain plays an important role in controlling growth, development, reproduction, and adaptation to environmental change. However, few studies stem from the perspective of whole transcriptome change in a fish brain and its response to long-term hypersaline stress. This study compares the differential transcriptomic responses of juvenile Nile tilapia (*Oreochromis niloticus*) maintained for 8 weeks in brackish water (16 practical salinity units, psu) and in freshwater. Fish brains from each treatment were collected for RNA-seq analysis to identify potential genes and pathways responding to hypersaline stress. A total of 27,089 genes were annotated, and 391 genes were expressed differently in the salinity treatment. Ten pathways containing 40 differentially expressed genes were identified in the tilapia brain. Antigen processing and presentation and phagosome were the two principally affected pathways in the immune system. Thirty-one of 40 genes were involved in various expressions associated with environmental information processing pathways such as neuroactive ligand-receptor interaction, cytokine-cytokine receptor interaction, the Jak-STAT signaling pathway, cell adhesion molecules (CAMs), and the PI3K-Akt signaling pathway, which are the upstream pathways for modulation of immunity and osmoregulation. The most-changed genes (>5-fold) were all down-regulated, including four growth hormone/prolactin gene families, i.e., prolactin precursor (−10.62), prolactin-1 (−11), somatotropin (−10.15), somatolactin-like (−6.18), and two other genes [thyrotropin subunit beta (−7.73) and gonadotropin subunit beta-2 (−5.06)] that stimulated prolactin release in tilapia. The downregulation pattern of these genes corroborates the decrease in tilapia immunity with increasing salinity and reveals an adaptive mechanism of tilapia to long-term hypersaline stress. Ovarian steroidogenesis, isoquinoline alkaloid biosynthesis, and phenylalanine metabolism are the three important pathways in the response of the fish to long-term hypersaline stress. This study has identified several pathways and relevant genes that are involved in salinity regulation in a euryhaline fish and provides insight into understanding regulatory mechanisms of fish to salinity change.

## Introduction

Osmoregulation is the ability of fish to adapt to a change in ambient salinity and is a complex process that has been studied extensively (Tseng and Hwang, 2008; Yousefian and Shirzad, 2011; Whittamore, 2012). The change in environmental osmolality has profound effects on fish at molecular, cellular and whole organismal levels, and fish need to make adaptive changes to maintain physiological functions and make compensatory adjustments for the change in habitat (Tseng and Hwang, 2008). In the process of osmoregulation, osmosensors in fish initially perceive osmotic stress, and then pass osmosensory signals to the brain before producing any response to alleviate osmotic stress (Kültz, 2012a,b). However, the majority of studies on this topic focus on osmosensory effectors and relevant metabolism processes, such as ion and water transport during osmoregulation, but knowledge regarding osmosensing and osmotic stress signal transduction on a molecular level is quite limited.

The fish brain, particularly the hypothalamus, and pituitary gland are important in osmotic homeostasis (Bernier et al., 2009). The variation in ambient salinity can lead to a change in fish plasma osmolality, which provokes changes in extracellular fluid surrounding brain cells (Abbott et al., 2010). Thus, fish brains contain sensitive target tissues to thoroughly explore the osmosensors and osmosensory signal transductor for osmoregulation. The regulation and signaling mechanisms involved in osmoregulation in fish brains have been studied under both hypo- and hyper-osmotic stress (Manzon, 2002; Gardell et al., 2013; Aruna et al., 2014; Kültz, 2015). Apart from brains, other recent studies performed on gilt-head seabream, *Sparus aurata*, showed a clear activation of pathways related to osmoregulation in the liver and gills after hypo- and hyper-osmotic challenges by means of a microarray approach (Martos-Sitcha et al., 2016). The existing literature focuses on several specific metabolites, genes or specific pathways, but information is limited regarding integral adaptive pathways to salinity in fish.

Nile tilapia, *Oreochromis niloticus*, is a species in aquaculture around the world, and rearing Nile tilapia in brackish water has received considerable attention in the past decade because of tilapia's wide range of salinity tolerance after appropriate salinity acclimation (Gan et al., 2016). As a result, various studies have been conducted on tilapia to explore the effects of salinity on fish osmoregulation (Al-Harbi and Uddin, 2005; Putra et al., 2013; Ninh et al., 2014; Thoa et al., 2015; Mashaii et al., 2016). Previous studies have shown that Nile tilapia can be reared in brackish water, but there are concerns regarding its slow growth, low immunity, and higher disease susceptibility (Chang and Plumb, 1996; Iqbal et al., 2012; Pereira et al., 2016; Bosisio et al., 2017). The underlying mechanism for how salinity can modulate fish growth and immunity has not been fully studied. Therefore, the purpose of this study is to reveal the brain transcriptome differences between Nile tilapia in brackish water and freshwater with RNA-seq technology. The results will provide new insight into the understanding of the integral adaptive pathways to salinity stress in Nile tilapia and other homologous species.

## Materials and methods

### Experimental animals, design, and sampling

The sex-reversed, all-male Nile tilapia juveniles were obtained from a private hatchery in Shenzhen, Guangdong, and were then acclimated for a week in the Aquaculture Nutrition and Environmental Health laboratory in East China Normal University. Prior to beginning the experiment, tilapia (6.41 ± 0.09 g) were randomly stocked into six tanks (66 × 63 × 40 cm) at a density of 18 fish per tank, which were independent aquariums with aerating apparatus. Three tanks were filled with freshwater, and then the remaining three were gradually changed to brackish water (16 practical salinity units, psu) by adding sea salt at an increasing rate of 4 psu per day. All fish were maintained in these tanks for 49 days prior to sampling. During this period, tilapia were fed to satiation with commercial feed twice daily (08:00 and 15:00 h), the remaining feed and feces were siphoned out and 1/3 of the water in each tank was replaced with pre-aerated water each day. Throughout the trial, water was continuously aerated, the photoperiod was maintained at 12 h light and 12 h dark, and water quality parameters were monitored. Dissolved oxygen was 7.7–8.9 mg/L, pH averaged 8.06 ± 0.23, ammonia nitrogen was <0.2 mg/L and water temperature averaged 27 ± 2°C. At the end of the trial, three fish from each tank were randomly selected and anesthetized with 100 ppm tricaine methanesulfonate (MS-222), and the brain was sampled for RNA extraction and RNA-seq analysis. All samples were frozen with liquid nitrogen and then stored at −80°C until analysis. The animal ethics protocol was approved by the East China Normal University Experimental Animal Ethics Committee (No. F20140101).

### RNA extraction

Total RNA was extracted from the tilapia brains using TRIzol® Reagent following the instructions (Invitrogen), and genomic DNA was removed with DNase I (TaKara). Subsequently, RNA quality was determined with a 2100 Bioanalyzer (Agilent) and quantified using a NanoDrop 2000 (NanoDrop Technologies). Only high-quality RNA samples (OD260/280 ranged 2.07–2.10, RIN ranged 9.04–9.70) were used to construct the sequencing library. Three qualified RNA samples from each tank were pooled with an equal amount of RNA. In total, there were six pooled RNA samples with three replicates for each group.

### Library preparation and Illumina HiSeq 4000 sequencing

The tilapia brain transcriptome library was prepared following the instructions in the TruSeq™ RNA sample preparation kit (Illumina) using 1 μg of brain RNA. In short, messenger RNA was isolated according to the polyA selection method, and then RNA was fragmented with the fragmentation buffer. Subsequently, double-stranded cDNA was synthesized using a SuperScript double-stranded cDNA synthesis kit (Invitrogen, CA) and random hexamer primers (Illumina). The synthesized cDNA was then subjected to end-repair, phosphorylation and “A” base addition, according to Illumina's library construction protocol. Libraries were size selected for the 200–300 bp cDNA target fragments on 2% agarose gel, following PCR amplification using Phusion DNA polymerase (NEB) for 15 PCR cycles. After being quantified with TBS380, the paired-end RNA-seq sequencing library was sequenced with an Illumina HiSeq 4000 (2 × 150 bp read length).

### RNA-seq raw data quality control and mapping

The programs SeqPrep (https://github.com/jstjohn/SeqPrep) and Sickle (https://github.com/najoshi/sickle) were used to remove low-quality reads (i.e., *Q*-values < 20), adapter sequences, reads with the ratio of ambiguous bases (“N”)>10%, and fragments <20 bp in length. The high-quality trimmed sequences were used for further mapping of the tilapia genome (GenBank accession No. 8126) with HISAT 2 (Trapnell et al., 2009, 2014).

### Differential expression analysis and functional enrichment

The expression level of each transcript was calculated to identify differential expression genes between the two different treatments, and the fragments per kilobase of exon per million mapped reads (FRKM) method was used in this study. RSEM (http://deweylab.biostat.wisc.edu/rsem/; Li and Dewey, 2011) was used to quantify gene abundance. Differential expression analysis was conducted with the R statistical package software EdgeR (Empirical analysis of Digital Gene Expression in R, http://www.bioconductor.org/packages/2.12/bioc/html/edgeR.html; Robinson et al., 2010). The differential gene screening criteria were fold change >2 or fold change <0.5 [*P* < 0.05, false positive rate (FDR) < 0.05]. If multiple transcripts existed in a gene, the longest transcript was selected to calculate the sequencing depth and expression to ensure result accuracy. Go and Kyoto encyclopedia of genes and genomes database (KEGG) were used for functional enrichment analysis to identify the differential expression genes that were involved in differently enriched metabolic pathways. Thus, the differentially expressed genes in significantly enriched GO terms were selected at *P* < 0.01 and FDR < 0.05. GO functional enrichment and KEGG pathway analysis were conducted with Goatools (https://github.com/tanghaibao/Goatools) and KOBAS (https://david.ncifcrf.gov/summary.jsp; Xie et al., 2011).

### Gene co-expression networks

The gene co-expression networks for 40 significantly changed genes involved in the significantly changed pathways were built according to their normalized expression values (FPKM). A Pearson correlation coefficient was calculated for each pair of genes, and the significant correlation pairs (*P* < 0.05, Pearson >0.8 or < -0.8) were selected to construct the network. Within the network analysis, degree centrality was the simplest and most important measure of the centrality of a gene within a network for determining the relative importance.

## Results

### Characteristics of the RNA-seq data

ILLUMINA data (Illumina MiSeq) was submitted to SRA on the NCBI website. The SRA Accession No. was SRP126457. A total of 352.65 million reads were obtained, including 54.52–60.62 million reads from tilapia in freshwater and 57.88–63.26 million reads in brackish water (Table 1). After filtration, a total of 344.34 million reads (97.64%) were generated for subsequent analysis, including reads from freshwater cultured tilapia ranging from 53.24 (97.66%) million to 58.86 (97.68%) million and reads from brackish water cultured tilapia ranging from 56.63 (97.81%) to 61.96 (97.93%) million. The uniquely mapped reads were 48.68 (91.44%) to 54.03 (91.80%) million in tilapia in freshwater and 51.96 (91.75%) to 56.87 (91.75%) million in tilapia in brackish water with a salinity of 16 psu.

**Table 1:** Summary statistics of the RNA-seq data of brains from Nile tilapia grown for 49 days in freshwater and brackish water with a salinity of 16 psu.

**Parameters**	**Freshwater (Control)**			**Brackish water (salinity of 16 psu)**			**Total**	**Average**
	**1**	**2**	**3**	**1**	**2**	**3**		
Total reads (× 10^6^)	54.52	58.83	60.26	57.90	63.26	57.88	352.65	58.775
Total reads after trimming (× 10^6^)	53.24	56.97	58.86	56.63	61.96	56.68	344.34	57.39
Reads filtered (%)	97.66	96.84	97.68	97.81	97.93	97.92	97.64	97.64
Total base (bp, × 10^9^)	8.23	8.88.	9.10	8.74	9.55	8.74	53.24	8.87
Total base after trimming (bp, × 10^9^)	7.83	8.33	8.66	8.33	9.12	8.34	50.61	8.435
Base filtered (%)	95.10	93.75	95.13	95.31	95.45	95.47	95.06	95.04
Mapped reads (× 10^6^)	48.68	52.42	54.03	51.96	56.87	52.01	315.97	52.66
Mapping rate (%)	91.44	92.01	91.80	91.75	91.79	91.75	91.76	91.76

### Differentially expressed genes

**Figure 1:**
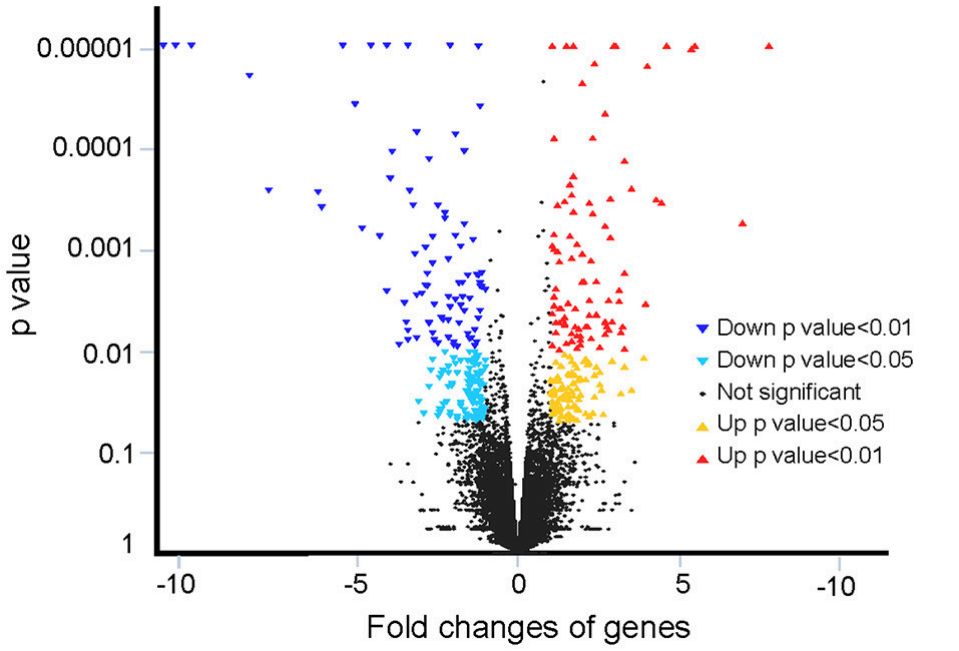
Overview of fold changes in gene expression in this study. The y-axis indicates the statistical *p*-value, and the x-axis indicates the fold changes of genes.

Gene identification was based on the Nile tilapia genome. A total of 27,089 genes were annotated, in which a total of 391 genes were differently expressed with a fold change >2 or < 0.5 (*P* < 0.05, FDR < 0.05) in the brain gene set for tilapia in brackish water vs. the control fish in freshwater (Figure 1).

### Significantly changed pathway analysis

**Figure 2:**
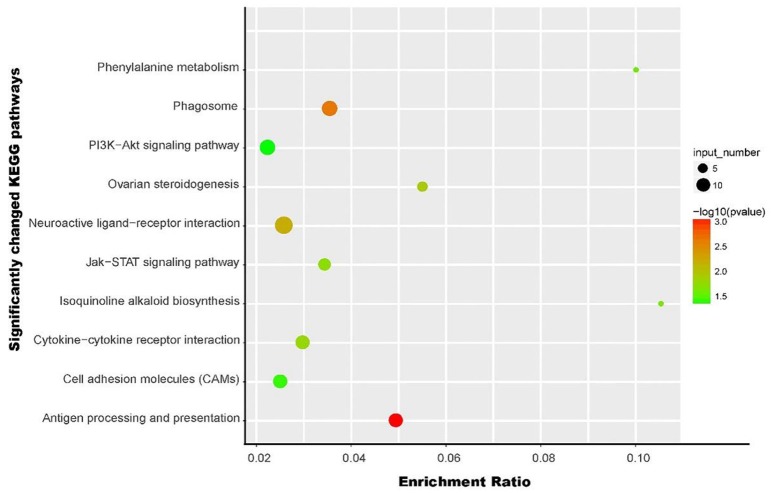
An overview of the significantly affected pathways from KEGG analysis in this study. The y-axis indicates specific pathways, and the x-axis indicates the enrichment ratio. The size of the colored dots indicates the number of significantly changed genes involved into each corresponding pathway, and pathways with larger-sized dots contain a higher number of genes. The color of the dots shows the –log *p*-value of the pathways.

Ten significantly changed pathways (*p* < 0.05) containing the differentially expressed genes were obtained using KEGG (Figure 2), and other changed pathways with no significant difference were shown in Supplementary Table 1. Five of the 10 pathways, including neuroactive ligand-receptor interaction, cytokine-cytokine receptor interaction, the Jak-STAT signaling pathway, CAMs, and PI3K-Akt signaling pathway, were the pathways of environmental information processing in Type I, and these five pathways were classified into either signaling molecules and interaction or signal transduction in Type II (Table 2). The most-changed pathway, antigen processing and presentation, belonged to the immune system, followed by the phagosome pathway belonging to transport and catabolism in Type II. Additionally, four genes were involved in the pathway in the endocrine system and ovarian steroidogenesis. The gene for primary amine oxidase liver isozyme-like isoform X1 and the gene for l-amino-acid oxidase-like isoform X1 were both involved in the two metabolism pathways for isoquinoline alkaloid biosynthesis and phenylalanine metabolism.

**Table 2:** Significantly changed pathways obtained with KEGG, using the significantly changed genes from brains of Nile tilapia grown for 49 days in brackish water with a salinity of 16 psu vs. those of tilapia in freshwater.

**Pathways**	**ID**	**Sample number**	**Background number**	***P*-value**	**Genes**	**Type II**	**Type I**
Antigen processing and presentation	ko04612	8	163	0.001	19932|2770|3482|2754|3460 (up-regulated) 20042|1466|18447 (down-regulated)	Immune system	Organismal systems
Phagosome	ko04145	10	285	0.002	3482|2754|3460|19932|2770 (up-regulated) 14114|1466|18447|20042|12747 (down-regulated)	Transport and catabolism	Cellular processes
Neuroactive ligand-receptor interaction	ko04080	14	552	0.006	24421|14071|5826|5357|6467|4654|1493|6469|9191 (up-regulated) 25798|16886|18430|7953|16259 (down-regulated)	Signaling molecules and interaction	Environmental information processing
Ovarian steroidogenesis	ko04913	4	73	0.012	26630|1493|2608|5826 (up-regulated)	Endocrine system	Organismal systems
Cytokine-cytokine receptor interaction	ko04060	8	272	0.016	6469|5357|6467|9191|11230 (up-regulated) 8910|18024|4273 (down-regulated)	Signaling molecules and interaction	Environmental information processing
Jak-STAT signaling pathway	ko04630	6	176	0.019	5357|6467|9191|6469 (up-regulated) 18024|4273 (down-regulated)	Signal transduction	Environmental information processing
Isoquinoline alkaloid biosynthesis	ko00950	2	19	0.024	14747|11903 (down-regulated)	Biosynthesis of other secondary metabolites	Metabolism
Phenylalanine metabolism	ko00360	2	20	0.026	14747|11903 (down-regulated)	Amino acid metabolism	Metabolism
Cell adhesion molecules (CAMs)	ko04514	8	324	0.039	3482|1472|19932 (up-regulated) 13988|15142|1466|18447|15920 (down-regulated)	Signaling molecules and interaction	Environmental information processing
PI3K-Akt signaling pathway	ko04151	10	455	0.044	6469|9191|6467 (up-regulated) 2865|14114|7953|141|2145|1075|5357 (down-regulated)	Signal transduction	Environmental information processing

### Key genes and their co-expression network analysis

**Figure 3:**
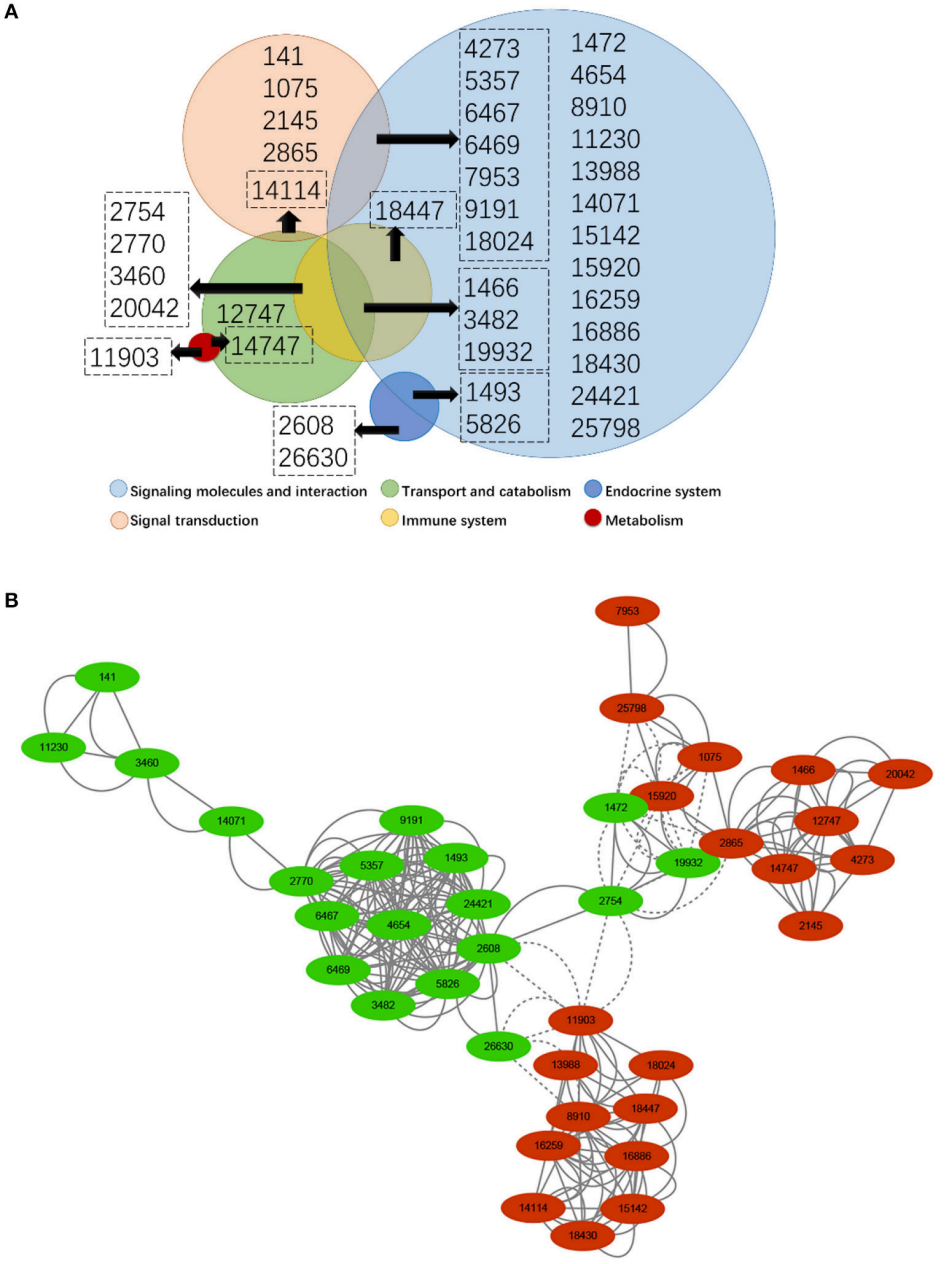
Analysis of genes involved in different pathways under various categories **(A)** and co-expression network of significantly changed genes **(B)**. In **(A)**, the circles with different colors show the pathways in different categories, and the circle size is positively correlated with the number of genes in each corresponding category. In **(B)**, green indicates down-regulated genes, while red indicates up-regulated genes. The dot size is positively correlated with the fold change of genes. Values inside all circles and dots are the number of genes shown in Table 3.

Forty of the 391 differentially expressed genes were involved in 10 significantly changed pathways, including 19 up-regulated and 21 down-regulated genes (Table 3), and many of the genes co-existed in several pathways. Thirty-one of the 40 genes were involved in the pathways related to signaling molecules and interaction (26 genes) and signal transduction (12 genes) in the environmental information processing of Type I (Figure 3A). Figure 3B shows the co-expression network of the 40 genes, and the 40 genes were clustered into four groups overall according to the expression, which proved difficult for further analysis due to complexity.

**Table 3:** Significantly changed genes and KEGG pathways in the brains of Nile tilapia grown for 49 days in brackish water vs. in freshwater.

**Seq_id**	**logFC(A/B)**	***P*-value**	**KEGG gene**	**Full name**
**ENVIRONMENTAL INFORMATION PROCESSING**				
**Signaling molecules and interaction**				
ENSONIG00000001466	1.57	1.76E-02	MHC1	Major histocompatibility complex class I-related gene protein-like
ENSONIG00000001472	−1.39	8.17E-04	MAG, GMA, SIGLEC4	Sialic acid-binding Ig-like lectin 10-like
ENSONIG00000001493	−5.06	3.79E-05	LHB	Gonadotropin subunit beta-2
ENSONIG00000003482	−3.47	5.30E-03	MHC1	H-2 class I histocompatibility antigen
ENSONIG00000004273	1.46	2.84E-02	IL10RB	Interferon alpha/beta receptor 1-like isoform X1
ENSONIG00000004654	−1.29	2.54E-02	GZMA	Granzyme K-like
ENSONIG00000005357	−6.18	2.78E-04	GH	Somatolactin-like
ENSONIG00000005826	−3.28	3.72E-04	FSH	FSH beta subunit precursor
ENSONIG00000006467	−10.62	3.18E-06	PRL	Prolactin precursor
ENSONIG00000006469	−11	8.13E-06	PRL	Prolactin-1
ENSONIG00000007953	2.19	4.45E-02	LPAR6, P2RY5	Lysophosphatidic acid receptor 6-like
ENSONIG00000008910	1.51	2.61E-02	TNFRSF14, HVEM	Tumor necrosis factor receptor superfamily member 5-like
ENSONIG00000009191	−10.15	6.21E-06	GH	Somatotropin
ENSONIG00000011230	−2.19	1.27E-03	IL1B	Interleukin-1 beta-like
ENSONIG00000013988	1.39	2.28E-02	PTPRF, LAR	Receptor-type tyrosine-protein phosphatase F-like
ENSONIG00000014071	−1.7	1.10E-04	DRD3	D(3) dopamine receptor-like isoformX2
ENSONIG00000015142	1.09	2.41E-02	PVRL1	Poliovirus receptor-related protein 2-like
ENSONIG00000015920	1.04	4.34E-03	VCAN, CSPG2	Versican core protein
ENSONIG00000016259	2.69	5.28E-03	TAAR	Trace amine-associated receptor 7g
ENSONIG00000016886	1.08	2.70E-02	CYSLTR1	Cysteinyl leukotriene receptor 1-like
ENSONIG00000018024	1.05	4.20E-02	IL22, IL-TIF	IL-22
ENSONIG00000018430	3.24	5.84E-03	CHRNB1	Acetylcholine receptor subunit beta-like
ENSONIG00000018447	2.83	7.73E-04	MHC1	Major histocompatibility complex class I
ENSONIG00000019932	−1.97	7.47E-04	MHC2	H-2 class II histocompatibility antigen
ENSONIG00000024421	−7.73	2.66E-04	TSHB	Thyrotropin subunit beta
ENSONIG00000025798	1.69	3.55E-02	P2RY14	Uncharacterized protein
**Signal transduction**				
ENSONIG00000000141	−1.68	4.12E-03	COL9A	Collagen alpha-1(IX) chain
ENSONIG00000001075	1.52	1.70E-02	COL4A	Collagen alpha-4(IV) chain-like isoform X1
ENSONIG00000002145	1.9	2.74E-02	GNG12	Guanine nucleotide-binding protein G(I)/G(S)/G(O) subunit gamma-12-like
ENSONIG00000002865	1.26	1.34E-03	LAMA3_5	Laminin subunit alpha-3-like
ENSONIG00000004273	1.46	2.84E-02	IL10RB	Interferon alpha/beta receptor 1-like isoform X1
ENSONIG00000005357	−6.18	2.78E-04	GH	Somatolactin-like
ENSONIG00000006467	−10.62	3.18E-06	PRL	Prolactin precursor
ENSONIG00000006469	−11	8.13E-06	PRL	Prolactin-1
ENSONIG00000007953	2.19	4.45E-02	LPAR6, P2RY5	Lysophosphatidic acid receptor 6-like
ENSONIG00000009191	−10.15	6.21E-06	GH	Somatotropin
ENSONIG00000014114	2.69	5.96E-04	TLR2	Toll-like receptor 2
ENSONIG00000018024	1.05	4.20E-02	IL22, IL-TIF	IL-22
**CELLULAR PROCESSES**				
**Transport and catabolism**				
ENSONIG00000001466	1.57	1.76E-02	MHC1	Major histocompatibility complex class I-related gene protein-like
ENSONIG00000002754	−1.26	1.13E-02	CTSS	Cathepsin S-like
ENSONIG00000002770	−1.24	1.39E-02	CTSS	Cathepsin K-like
ENSONIG00000003460	−1.28	4.99E-02	CTSL	Cathepsin L1
ENSONIG00000003482	−3.47	5.30E-03	MHC1	H-2 class I histocompatibility antigen
ENSONIG00000012747	1.11	2.95E-03	TUBA	Interferon-induced protein 44-like isoform X3
ENSONIG00000014114	2.69	5.96E-04	TLR2	Toll-like receptor 2
ENSONIG00000018447	2.83	7.73E-04	MHC1	Major histocompatibility complex class I
ENSONIG00000019932	−1.97	7.47E-04	MHC2	H-2 class II histocompatibility antigen
ENSONIG00000020042	2.79	6.26E-03	ABCB3, TAP2	Antigen peptide transporter 2-like
**ORGANISMAL SYSTEMS**				
**Immune system**				
ENSONIG00000001466	1.57	1.76E-02	MHC1	Major histocompatibility complex class I-related gene protein-like
ENSONIG00000002754	−1.26	1.13E-02	CTSS	Cathepsin S-like
ENSONIG00000002770	−1.24	1.39E-02	CTSS	Cathepsin K-like
ENSONIG00000003460	−1.28	4.99E-02	CTSL	Cathepsin L1
ENSONIG00000003482	−3.47	5.30E-03	MHC1	H-2 class I histocompatibility antigen
ENSONIG00000018447	2.83	7.73E-04	MHC1	Major histocompatibility complex class I
ENSONIG00000019932	−1.97	7.47E-04	MHC2	H-2 class II histocompatibility antigen
ENSONIG00000020042	2.79	6.26E-03	ABCB3, TAP2	Antigen peptide transporter 2-like
**Endocrine system**				
ENSONIG00000001493	−5.06	3.79E-05	LHB	Gonadotropin subunit beta-2
ENSONIG00000002608	−1.22	4.84E-02	CYP2J	Cytochrome P450 2J2
ENSONIG00000005826	−3.28	3.72E-04	FSH	FSH beta subunit precursor
ENSONIG00000026630	−1.06	3.76E-02	CYP2J	Cytochrome P450 2J2-like
**Metabolism**				
ENSONIG00000011903	1.27	3.90E-03	AOC3, AOC2, tynA	Primary amine oxidase, liver isozyme-like isoform X1
ENSONIG00000014747	1.77	1.39E-02	IL4I1	L-amino-acid oxidase-like isoform X1

## Discussion

Hypersaline stress can exert various adverse effects on Nile tilapia, including slow growth, low disease resistance, and impaired immune and antioxidant functions (Chang and Plumb, 1996; Iqbal et al., 2012; Gan et al., 2016; Pereira et al., 2016; Bosisio et al., 2017). The brain is the central organ controlling the behavior and physiology of the animal, and the response of a fish brain to environmental change may explain the adaptive mechanism of a fish to hypersaline stress. This initial study describes the transcriptomic response of the Nile tilapia brain to long-term hypersaline stress and reveals new insights into the understanding of the brain's regulative mechanism for osmoregulation.

In immune pathways, antigen processing and presentation and phagosome are the two pathways that were most affected by long-term hypersaline stress in the tilapia brain. Three genes for major histocompatibility complex (MHC) class I, related protein-like, and antigen peptide transporter 2-like were up-regulated in both of these two pathways. Additionally, the interferon-induced protein 44-like isoform X3, as the precursor to the histocompatibility antigen HA-28, and the toll-like receptor 2, as an important protein in the innate immune system of a fish (Palti, 2011) in the phagosome pathways, were up-regulated. The MHC class I antigen presentation pathway is one of the general distinct pathways for presenting peptide antigens to CD8+ and CD4+ T cells and is active in almost all cell types by providing cell surface proteins essential for the acquired immune system to recognize foreign molecules in vertebrates (Jensen, 2007). Therefore, the increased gene expression for MHC class I related proteins or antigen peptide transporters might be related with the acquired immunity of tilapia to long-term hypersaline stress. However, two other genes in MHC for the H-2 class I histocompatibility antigen and the H-2 class II histocompatibility antigen and three genes for cathepsin S-like, cathepsin K-like, and cathepsin L1 were down-regulated in both pathways of phagosomes and antigen processing and presentation. Cathepsins are proteases responsible for lysosome protein degradation and are widely distributed among prokaryotes and eukaryotes, in which cathespins play an immune function in fish and other vertebrates (Harikrishnan et al., 2010; Sansri et al., 2015; Wang et al., 2016). The decreased expression of cathepsin genes indicated a reduction in lysosome degradation. Cells may exhibit a high demand for lysosome function under salinity stress, which is why the degradation of lysosome decreased in this study. However, further research is still necessary to confirm this inference.

Although tilapia has the potential to tolerate certain levels of salinity (Nugon, 2003), a salinity above 8 psu can pose adverse effects on the immune response, disease resistance and antioxidant capacity in tilapia (Alsaid et al., 2013; Dominguez et al., 2015; Gan et al., 2016; Qiang et al., 2016). In this study, we also determined that long-term hypersaline stress will cause disorders in the immune systems of Nile tilapia. Therefore, from a practical standpoint, considerable work, such as nutritional modulation, should be further conducted to solve this problem.

Various adverse effects on Nile tilapia in hypersaline water, including impaired immune capacity and slow growth, are closely related to fish brain modulation. In this study, 5 of the 10 significantly changed pathways were related to environmental information processing, including neuroactive ligand-receptor interaction, cytokine-cytokine receptor interaction, CAMs in the category of signaling molecules and interaction, and the Jak-STAT signaling pathway and the PI3K-Akt signaling pathway in the signal transduction category (Table 2). The neuroactive ligand-receptor interaction pathway comprised all ligands and receptors in the cell membrane for signal transduction (Lauss et al., 2007) and was largely changed by salinity stress. The cytokine-cytokine receptor interaction pathway has crucial effects on inflammation in animals, and its dysfunction has been used to diagnose a variety of pathological changes (Dey et al., 2009; Lasry et al., 2016). CAMs are proteins located on the cell surface, and their functions include assembly and interconnection of various cellular functions, maintenance of tissue integration, and wound healing (Chi and Melendez, 2007; Dustin, 2007). The Jak-STAT signaling pathway transmits information from extracellular chemical signals to the nucleus, resulting in DNA transcription and expression of genes involved in immunity, proliferation, differentiation, and cell apoptosis (Aaronson and Horvath, 2002). Our results show that the neurotransmitters and receptors in fish brains are very important for sensing ambient salinity stress and signaling in modulating fish physiology (Aruna et al., 2012, 2014; Martins et al., 2013; Upton and Riley, 2013). Additionally, in the current study, most of the changed pathways are related to animal immunology; therefore, the low immune capacity of Nile tilapia under hypersaline stress might be an outcome of these responsive pathways. Furthermore, the PI3K-Akt signaling pathway, the least significantly changed pathway, is one of the most actively studied kinase pathways as it plays an integral role in mediating signals for cell growth, survival, cell-cycle progression, differentiation, transcription, translation, and glucose metabolism in animals (Yao et al., 2014; Ferreira et al., 2016; Li et al., 2017). Therefore, the downregulation of this pathway could be the reason for slowed growth of Nile tilapia under hypersaline conditions (Gan et al., 2016), since fish require more energy during normal growth for osmoregulation and icon homeostasis (Tseng and Hwang, 2008).

Thirty-one genes with various expressions were involved in the pathways in the brain of Nile tilapia and were related to signaling molecules and interaction and signal transduction. However, changes >5-fold were only observed in six down-regulated genes, namely prolactin precursor (−10.62), prolactin-1 (−11), somatotropin (−10.15), somatolactin-like (−6.18), thyrotropin subunit beta (−7.73), and gonadotropin subunit beta-2 (−5.06). Prolactin is a fundamental endocrine factor for hyper-osmoregulation in teleost fishes. Prolactin in the gills can maintain the ion concentrations of body fluid within a narrow physiological range in freshwater conditions. Previous studies have shown that prolactin directly decreases the permeability of gill epithelia in trout (Kelly and Wood, 2002). Therefore, the significant downregulation of prolactin and its precursor indicates that fish can increase cell permeability to adapt to hypersaline stress, and extra ion intake can be efficiently expelled outside the body.

The immune-stimulatory effect of prolactin is associated with the expressions of toll-like receptors (Peã et al., 2016; Soto et al., 2016), macrophage function (Paredes et al., 2013), and immunoglobulin M production in the fish humoral immune system (Yada et al., 1999). Somatotropin and somatolactin-like genes, together with prolactin, all belong to the growth hormone/prolactin family and play a central role in controlling growth (Kasper et al., 2006) and enhancing tilapia innate immunity (Acosta et al., 2009; Uchida et al., 2009). Additionally, thyrotropin-releasing hormone is a hypothalamic hypophysiotropic neuropeptide that can regulate the synthesis and release of prolactin. Gonadotropin-releasing hormones can stimulate the secretion of growth hormone, prolactin, and somatolactin in a particular stage of teleost development (Bhandari et al., 2003). In addition, the thyroidal system has been demonstrated to play an important role in osmoregulation in other euryhaline species, such as *Senegalese sole* and *S. aurata* (Arjona et al., 2011; Ruiz-Jarabo et al., 2016, 2017), and both of the species were involved in ion regulation and additionally in the stress pathway. Both thyrotropin subunit beta and gonadotropin subunit beta-2 were significantly down-regulated in this study. Thus, the overall dysfunction of the immune system and slow growth of Nile tilapia under hypersaline stress is partially caused by the downregulation of genes in the growth hormone/prolactin family and the corresponding regulation functions.

Under salinity stress, fish need to maintain internal osmotic and ionic homeostasis for normal cellular morphology and physiological functions and metabolism of various enzymes and transporters (Evans et al., 2005). In this study, the primary amine oxidase gene (liver isozyme-like isoform X1) and the L-amino-acid oxidase-like isoform X1 gene were up-regulated under hypersaline stress. These two genes are both categorized into the pathway of phenylalanine metabolism. Phenylalanine may play a role in the seawater acclimation process in tilapia, because several essential amino acids such as phenylalanine in the tilapia (*O. mossambicus*) plasma increased following transfer from freshwater to seawater at 34 psu for 24 h in a previous study (Vijayan et al., 1996). Although no detailed information has been reported regarding the function of a high phenylalanine concentration, essential amino acids may be required for the synthesis of peptides and proteins, and their availability may regulate the synthesis of hormones that are important in the ion regulation process (Rodgers et al., 1992).

The pathway of ovarian steroidogenesis was also affected by hypersaline stress, and the four genes involved in this pathway were all significantly down-regulated. The significant change of this pathway, together with other steroid metabolism related pathways, was also observed in the hepatopancreas transcriptome analysis of Nile tilapia acclimated to a high salinity of 16 psu (Xu et al., 2015), showing the importance of steroids in fish osmoregulation. The hepatopancreas transcriptome analysis shows that in a hyper-osmotic environment, the ovarian steroidogenesis pathway can activate the cAMP signal pathway and stimulate the activity of adenylate cyclase in tilapia hepatopancreases (Aronica et al., 1994). The cAMP signal pathway can stimulate the production of arachidonic acid metabolites, which can regulate hormone production, including cortisol, glucagon and hormones related to osmoregulation and cellular fatty acid signaling in tilapia (Aronica et al., 1994; Franzellitti et al., 2011).

## Conclusion

Brain transcriptome analysis was conducted on Nile tilapia in a salinity of 16 psu and in freshwater. In all, 391 genes and 10 pathways significantly changed as a consequence of adaptation to hypersaline stress. Forty of the 391 genes were involved in 10 pathways. Immune-related pathways, antigen processing and presentation, and phagosome are the principally changed pathways through gene downregulation and correspond to immunity dysfunction in tilapia under long-term hypersaline stress. Upstream pathways for signaling molecules and interactions and signal transduction are involved, not only in coping with hypersaline stress but also in modulating immune functions under hypersaline stress. The growth hormone/prolactin gene families together with the immune-related pathways are down-regulated by hypersaline stress. Ovarian steroidogenesis and metabolism pathways are also involved in the process of dealing with hypersaline stress in the Nile tilapia brain, but their functionality warrants further investigation.

## Author contributions

YL: Conducted the experiment and wrote the manuscript; EL: Design the experiment and, analyzed the data, and wrote the paper; CX: Sample analysis and paper writing; YS: Contributed to the cultivation of tilapia and analyzed experimental data in our work. JQ: Experiment design and paper revision; LC: Experiment design and data analysis; XW: Sample analysis and paper writing. YL, XW and EL: Revised the paper.

### Conflict of interest statement

The authors declare that the research was conducted in the absence of any commercial or financial relationships that could be construed as a potential conflict of interest.
